# Interference with pulse oximetry by the Stealth Station™ Image Guidance System

**DOI:** 10.1186/s40981-017-0076-7

**Published:** 2017-01-13

**Authors:** Junichi Saito, Masato Kitayama, Ryutaro Kato, Kazuyoshi Hirota

**Affiliations:** 1 Department of Anesthesiology, Hirosaki University Graduate School of Medicine, Zaifu-cho 5, Hirosaki, 036-8562 Japan; 2 Division of Operating Center, Hirosaki University Medical Hospital, Hirosaki, Japan; 3 Department of Clinical Engineering, Hirosaki University Medical Hospital, Hirosaki, Japan

**Keywords:** A pulse oximetry, Neuro-navigation system, The infrared light

## Abstract

**Background:**

A pulse oximeter is one of the most important monitors to save patients undergoing anesthesia and monitored sedation. The authors report a case of orthopedic surgery, in which interference of pulse oximetry occurred when using a Stealth Station™ navigation system (Medtronic Sofamor Danek, Memphis, TN). Applying a black plastic shield (Masimo Ambient Shield: Masimo Corporation, Irvine, CA) completely eliminated the interference.

**Case presentation:**

A 37-year-old male patient with a giant cell tumor of the left femur was scheduled to undergo curettage of the femur using an intraoperative CT three-dimensional imaging system (O-arm™) and Stealth Station™ navigation system. During the surgery, the SpO_2_ value, which was maintained between 97 and 99% until the time, disappeared suddenly with abnormal pulse wave. Because a distortion in the SpO_2_ value was reproduced by repeated movement of cameras on the head of the Stealth Station™ navigation system, we recognized that the interference signal was coming from the navigation system. To eliminate the infrared light, the pulse oximetry probe was covered with a black plastic shield and the interference was completely eliminated.

**Conclusions:**

The Stealth Station™ navigation system was found to interfere with the SpO_2_ value, and a black plastic shield was useful for eliminating the interfering signal. Anesthesiologists should understand the risk of interference by the neuro-navigation system and know how to solve the problem.

## Background

A pulse oximeter is one of the most important monitors to save patients undergoing anesthesia and monitored sedation. The American Society of Anesthesiologists [1] recommends using a pulse oximetry during anesthesia. Being accurate, simple, and the non-invasive nature during use are its advantages. However, some factors can interfere with the accuracy of the measurement and may result in erroneous readings, affecting the neuro-navigation system [2, 3]. Recently, the Stealth Station™ Image Guidance System (Medtronic Sofamor Danek, Memphis, TN) has been used more frequently during surgery, including neurosurgery, orthopedic, and otolaryngologic surgery. Therefore, when using a pulse oximetry, anesthesiologists should understand the risk of interference by the neuro-navigation system and know how to solve the problem.

The authors report a case of orthopedic surgery, in which interference of pulse oximetry occurred when using a Stealth Station™ navigation system. Applying a black plastic shield (Masimo Ambient Shield: Masimo Corporation, Irvine, CA) completely eliminated the interference.

## Case presentation

**Fig. 1:**
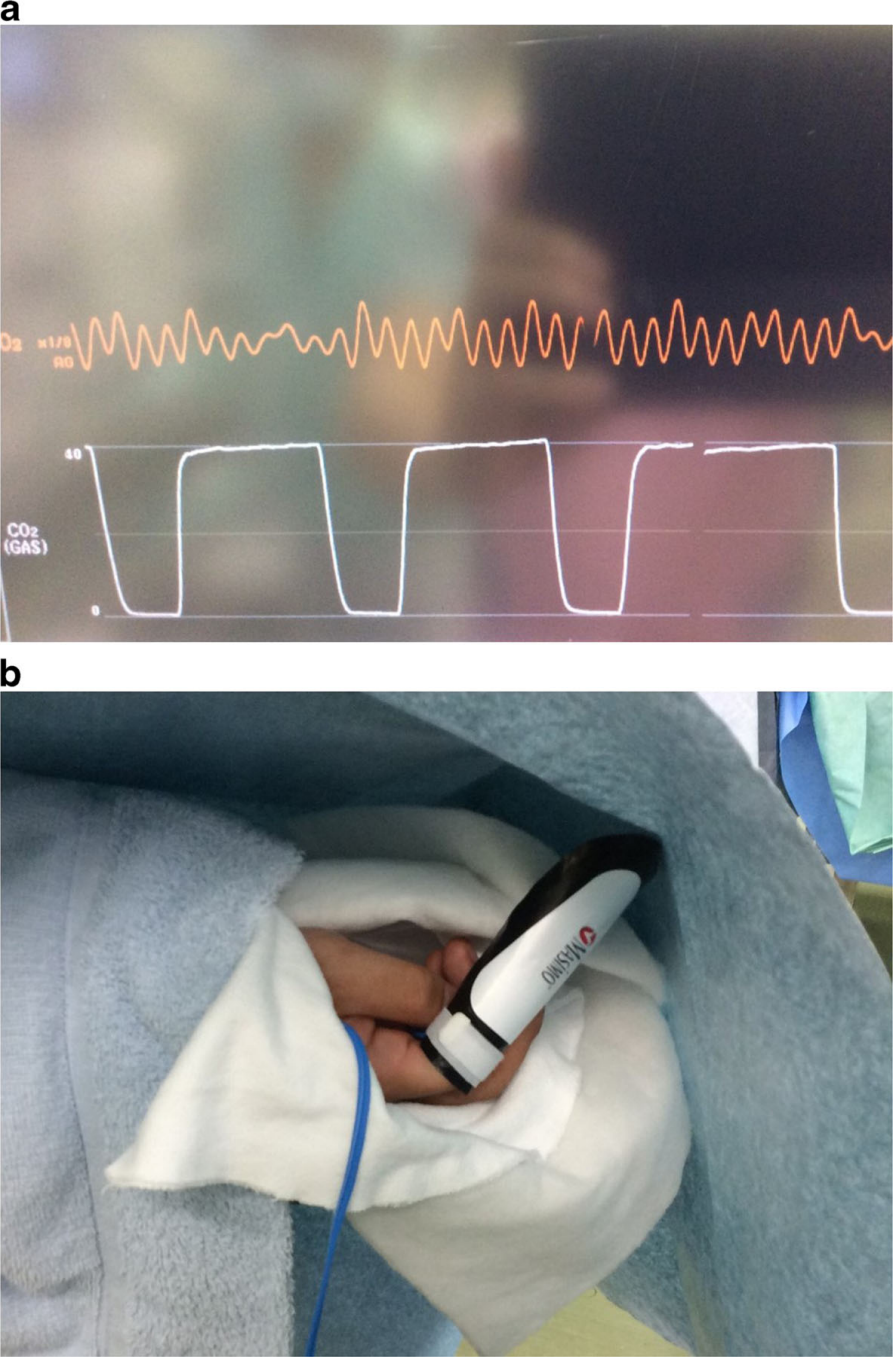
**a** Pulse oximetry plethysmogram with interference. **b** A black plastic shield to eliminate the interfering signal

A 37-year-old male patient with a giant cell tumor of the left femur was scheduled to undergo curettage of the femur using an intraoperative computed tomography three-dimensional imaging system (O-arm™) and Stealth Station™ navigation system. Throughout the procedure, the following variables were continuously monitored: electrocardiogram, peripheral oxygen saturation (SpO_2_) using a TL-273 SpO_2_ Probe™ (NIHON KOHDEN Corporation, Tokyo), end-tidal concentration of carbon dioxide (CO_2_), naso-esophageal temperature, and bispectral index. A small incision was made to expose the left femur. The patient reference frame was attached to the diaphysis of the femur to allow for optimal camera positioning and line of site. The SpO_2_ value, which was maintained between 97 and 99% until the time, disappeared suddenly with abnormal pulse wave (Fig. 1a). At first, the cause of interference was not recognized because the infrared (IR) light was not visible to the human eye. However, a distortion in the SpO_2_ value was reproduced by repeated movement of cameras on the head of the Stealth Station™ navigation system. Other possible causes for interference in SpO_2_, such as body movements, a disconnection, and an inadequate attachment of the pulse oximetry probe to the patient, were excluded. We recognized that the interference signal was coming from the Stealth Station™ navigation system. To eliminate the interference from the IR light, the pulse oximetry probe was covered with a black plastic shield (Fig. 1b) and the interference was completely eliminated.

### Discussion

We found out two important clinical issues. First, the Stealth Station™ navigation system can interfere with the SpO_2_ value. It is because pulse oximetry measurement is based on the red (R) and IR light absorption characteristics of the oxygenated and deoxygenated hemoglobin. After the R and IR signals pass through the measuring site and are received at the photo-detector, the R/IR ratio is calculated. The ratio converts to a SpO_2_ value. The Stealth Station™ navigation system emits a strong IR light, and the light from the navigation system interferes with the detection of IR light signals emitted from the pulse oximetry probe.

Second, a black plastic shield was successful in eliminating the interfering signal. To eliminate the IR light interference, shielding the pulse oximetry probe with aluminum was recommended [2, 3]. However, a black plastic shield, one of the accessories of Masimo Radical-7, is indicated for use with Masimo adhesive, re-usable and responsive sensor, to reduce ambient light interference. The black plastic shield could completely eliminate the strong IR light from influencing the navigation system. However, only for eliminating the IR light, this black plastic shield may be expensive. When aluminum foil cannot be used immediately or when repositioning the probe to other sites where the IR light does not reach pulse oximetry probe is difficult, this black plastic shield can be an alternative method to solve the IR light interference.

Not only the pulse oximetry but the mainstream capnograph also incorporates an IR sensor which measures the absorbance of IR light due to the presence of CO_2_. The electromagnetic interference from the Stealth Station™ navigation system might have interfered with the measurement in the capnograph leading to a misreading. Therefore, knowledge about the IR light-induced artifactual changes in intraoperative monitoring is important to prevent both diagnostic confusion and unnecessary interventions.

## Conclusions

The Stealth Station™ navigation system was found to interfere with the SpO_2_ value, and a black plastic shield was useful for eliminating the interfering signal.

## Abbreviations

CO_2_: Carbon dioxide
IR: Infrared
R: Red
SpO_2_: Peripheral oxygen saturation
